# Bayesian Mapping of HIV Infection among Women of Reproductive Age in Rwanda

**DOI:** 10.1371/journal.pone.0119944

**Published:** 2015-03-26

**Authors:** François Niragire, Thomas N. O. Achia, Alexandre Lyambabaje, Joseph Ntaganira

**Affiliations:** 1 Department of Applied Statistics, College of Business and Economics, University of Rwanda, Kigali, Rwanda; 2 School of Public Health, University of Witwatersrand, Johannesburg, South Africa; 3 Department of Human Nutrition and Dietetics, College of Medicine and Health Sciences, University of Rwanda, Kigali, Rwanda; 4 Department of Epidemiology and Biostatistics, College of Medicine and Health Sciences, University of Rwanda, Kigali, Rwanda; University of Perugia, ITALY

## Abstract

HIV prevalence is rising and has been consistently higher among women in Rwanda whereas a decreasing national HIV prevalence rate in the adult population has stabilised since 2005. Factors explaining the increased vulnerability of women to HIV infection are not currently well understood. A statistical mapping at smaller geographic units and the identification of key HIV risk factors are crucial for pragmatic and more efficient interventions. The data used in this study were extracted from the 2010 Rwanda Demographic and Health Survey data for 6952 women. A full Bayesian geo-additive logistic regression model was fitted to data in order to assess the effect of key risk factors and map district-level spatial effects on the risk of HIV infection. The results showed that women who had STIs, concurrent sexual partners in the 12 months prior to the survey, a sex debut at earlier age than 19 years, were living in a woman-headed or high-economic status household were significantly associated with a higher risk of HIV infection. There was a protective effect of high HIV knowledge and perception. Women occupied in agriculture, and those residing in rural areas were also associated with lower risk of being infected. This study provides district-level maps of the variation of HIV infection among women of child-bearing age in Rwanda. The maps highlight areas where women are at a higher risk of infection; the aspect that proximate and distal factors alone could not uncover. There are distinctive geographic patterns, although statistically insignificant, of the risk of HIV infection suggesting potential effectiveness of district specific interventions. The results also suggest that changes in sexual behaviour can yield significant results in controlling HIV infection in Rwanda.

## Introduction

Global disparities in the prevalence of human immunodeficiency virus (HIV) infection have been reported repeatedly [1–4]. It is estimated that 69% of all people living with HIV and acquired immune deficiency syndrome (AIDS) are in sub-Saharan Africa [3], where 1 in every 20 adults (4.9%) is living with HIV and the majority of them are women [2]. Within sub-Saharan Africa, differences in HIV infection levels have been found across and within countries, and range from less than 1% to more than 26% [4].

Rwanda is one of the sub-Saharan African countries where, after the first HIV-infected case was discovered in 1983, the virus spread rapidly throughout the country to reach generalized epidemic levels [5]. The national HIV prevalence rate within the adult population (15–49 years) was estimated as being as high as 11% in 2000, and then dropped to around 3.7% [2,6] in 2001. The national HIV prevalence rate has not decreased since the year 2005[7,8]. The 2005 and 2010 Rwanda demographic and health surveys (RDHS) estimated the national HIV prevalence rate among the adult population at 3% [8].

This national average prevalence rate also masks important within-country variations [8]. For example, total HIV prevalence is more than two times higher in urban (7.1%) than in rural (2.3%) areas. HIV prevalence among women is more than three times higher in the City of Kigali (9.4%) than the national average, while the prevalence in the other four provinces range from 2.5% to 3.2% [8].

In 2007, HIV prevalence among pregnant women attending antenatal care services fell slightly to 4.3% from an estimated 5.2% in 2003. In 2009, Rwanda was among countries with the largest number of pregnant women living with HIV [2]. The cross-sectional 2005 and 2010 RDHS reported adult women as having an HIV prevalence rate of 3.6% and 3.7% respectively [8]. In general, HIV prevalence in Rwanda has been consistently higher among women across all demographic and socioeconomic layers [7,8].

Therefore the control of HIV infection among women must be prioritised to prevent the transmission of the virus to uninfected men and to children during delivery or breastfeeding, which contributes more than 90% of pediatric HIV [9]. In addition, regardless of the offspring’s HIV status, there are further benefits in child survival as women are the primary child health care givers [10,11].

Nonetheless these benefits cannot be sustained in Rwanda if HIV prevalence among women keeps increasing. Factors explaining the reason HIV prevalence has remained higher among women are not well understood. Effective and efficient HIV prevention interventions presuppose the knowledge of the most influential factors that underlie HIV transmission differentials. Such knowledge is crucial for prioritising HIV interventions in Rwanda, where scarce resources are required in order to achieve the MDG 6 targets due for evaluation in 2015. With such knowledge intervention strategies become more sensitive to the cultural and socio-economic context of the intended community, and address local HIV transmission risk factors [12]. In addition, available resources are deployed where they will yield the highest impact [13].

A number of studies have investigated risk factors of HIV infection within and across various countries [12–24]. These studies have found specific and dissimilar risk factors with the result that country specific results cannot be reliably extrapolated over another country. Each of these studies found different sets of socio-economic [12–14,16–19,22], demographic [12,15,20,21], cultural [16], and behavioural [12,13,15–17,21–23] factors that are associated with the distribution of HIV infection. For example, socio-demographic and cultural factors such as age, marital status, mobility and religion have been associated with the risk of HIV infection [15,17,21,23]. Factors pertaining to household headship were also found important in countries recovering from armed conflicts where members of minor- or women-headed households were more likely to be at a higher risk [25]. Socio-economic factors such as educational attainment, household wealth, type of place of residence, employment, and exposure to mass media are among the reported background risk factors of HIV infection [13,14,19]. In addition, factors such as HIV/AIDS stigma and knowledge, age at first sexual experience, and the number of sexual partners have repeatedly been associated with the risk of HIV infection [21,26]. The occurrence and recurrence of sexually transmitted infections (STI) prepare an open route for HIV entry into the human body [26–30]. The geographic location where people live also provide measurable and immeasurable factors that shape individuals’ behaviour and beliefs as well as their socio-economic performance and hence influence the risk of HIV transmission [16,20,31].

Since more than 8 years, no strategy against HIV infection in Rwanda has resulted in a decline of the national HIV prevalence, which has stabilised at 3%. Therefore, there is a high demand for a more advanced analysis of available data to refine the current understanding of the epidemic in order to assist HIV prevention policy formulation.

This study investigates underlying and proximate risk factors as well as small-area spatial variation of HIV infection among women of reproductive age. The latter constitute one of the most at risk population groups in Rwanda, among whom HIV prevalence has been consistently higher and rising. It is hypothesised that HIV infection not only varies according to underlying and proximate factors but that it also varies across districts. This study aims to identify key determinants of HIV infection among women and to assess district-level spatial variation of HIV infection. It uses a unified, advanced and flexible statistical modelling framework that is capable of detecting small-area variations of spatial effect [20,32].

Until the year 2011 such a study was not possible in Rwanda because of data limitations. Although the 2005 RDHS was the first to collect HIV infection data, the 2006 administrative reform hindered such a study as the previous district boundaries were completely modified [33].

## Materials and Methods

### Study setting

This study is conducted in Rwanda, a mountainous country of 26338 square kilometres (sq.km). Rwanda is situated in central east Africa between latitude 1°4’ and 2°51' south and longitude 28°63' and 30°54’ east. It is a completely landlocked country surrounded by the Democratic Republic of Congo to the west, Uganda to the north, Tanzania to the east, and Burundi to the south [8]. The internal as well as international road networks are well maintained facilitating movements of persons and goods.

Rwanda is administratively subdivided into five provincial entities namely the City of Kigali, the Southern, the Northern, the Eastern, and the Western Province. In turn, provinces are subdivided into 30 districts, and 416 Sectors [33]. Table 1 lists the 30 districts of Rwanda according to their corresponding geographic codes used during the 2010 RDHS.

**Table 1:** Districts of Rwanda and their geographic codes used during the 2010 RDHS.

Code	District	Code	District	Code	District
1	Nyarugenge	11	Kamonyi	21	Musanze
2	Gasabo	12	Karongi	22	Burera
3	Kicukiro	13	Rutsiro	23	Gicumbi
4	Nyanza	14	Rubavu	24	Rwamagana
5	Gisagara	15	Nyabihu	25	Nyagatare
6	Nyaruguru	16	Ngororero	26	Gatsibo
7	Huye	17	Rusizi	27	Kayonza
8	Nyamagabe	18	Nyamasheke	28	Kirehe
9	Ruhango	19	Rulindo	29	Ngoma
10	Muhanga	20	Gakenke	30	Bugesera

**Fig 1:**
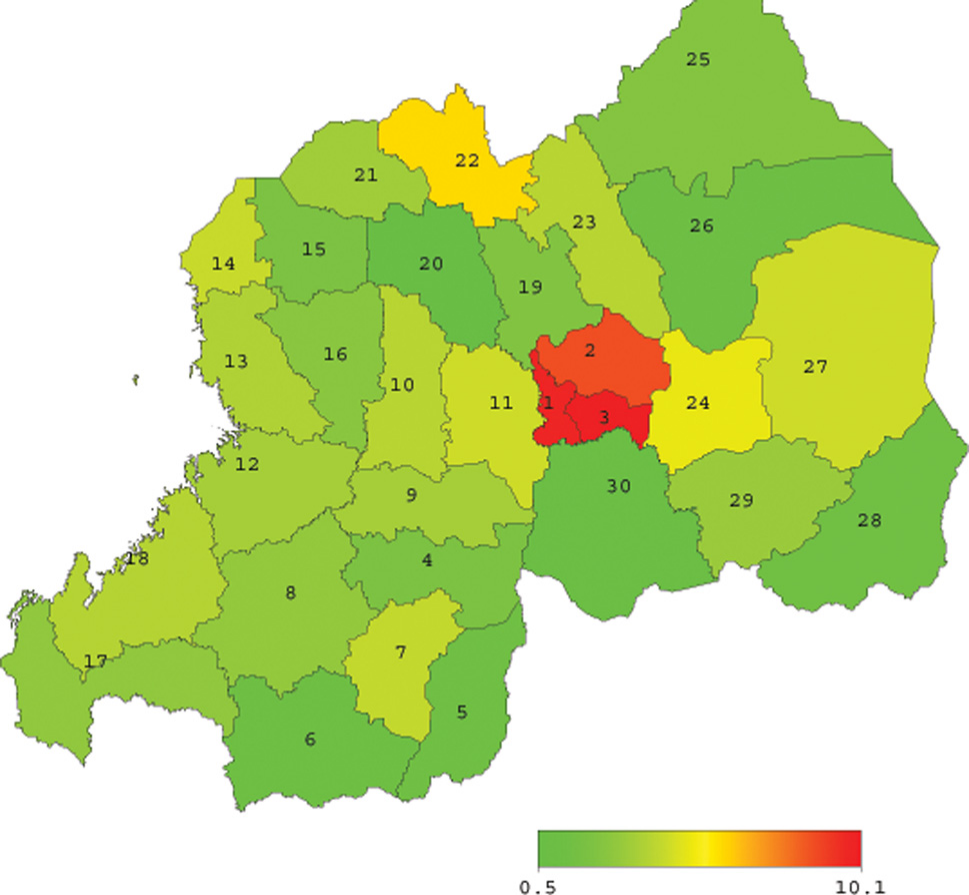
District codes and levels of HIV prevalence among women (15–49 years) in Rwanda.

Fig. 1 depicts the geospatial arrangement of the districts of Rwanda. Shown on the map is the district-level HIV prevalence rate among women as published by the 2010 RDHS [8], and the numbers shown on the map correspond to the district codes indicated in Table 1.

In the year 2012, the Rwandan population totalled 10,537,222 with an annual growth rate of 2.6%. The population is unevenly distributed throughout the country, and is predominantly rural depending mainly on subsistence agriculture [34]. The fertility rate was estimated as high as 4.6 children per woman in 2010, slowly declining from 6.1 in 2005 [8]. The population density has remained among the world’s highest and has increased from 321 in 2002 to 416 persons per sq.km in 2012 [34].

Rwanda has a low (<0,499) human development index (HDI) of 0.429, ranking 166^th^ out of 187 countries in 2011 on the Human Development Index. In 2011, Rwanda’s gross domestic product (GDP) per capita increased to USD 595 from USD 540 for the year 2010. In the year 2010, the proportion of the population living below the national poverty line dropped from 56.7% in 2005 to 44.9% [35].

### Data

The data used in this study come from the most recent Rwanda Demographic and Health Survey (RDHS) conducted in 2010 by the National Institute of Statistics of Rwanda (NISR). The 2010 RDHS is currently the only population-based and nationally representative sample survey in Rwanda that tested for HIV infection and collected large amount of diversified participant’s characteristics. Data were collected on the geographic location of each surveyed woman’s residence, household characteristics with a large set of behavioural, socioeconomic, and socio-demographic indicators together with individual woman’s HIV test result. Details about the 2010 RDHS multistage sample design, data collection methods, and data quality control and assurance are available in the 2010 RDHS report [8].

According to the same report, in total 12540 households were covered by the survey where 13671 women aged 15–49 were interviewed. Women from half of the surveyed households were also selected for HIV testing and 99% of them provided their consent [8].

In order to investigate the factors associated with the spread of HIV infection among women, the 2010 RDHS-based woman’s data file and HIV test results’ data file were obtained from MEASURE DHS, and were matched using relevant case identifier variables. Each woman’s HIV test status was matched with her socioeconomic, cultural, behavioural and demographic characteristics including her district of residence. Thus the matched data provided a very rich dataset which enabled, not only the assessment of factors associated with HIV transmission to women, but also the investigation of localised district-specific risk factors of HIV spread among women.

The matched dataset comprised 6592 women aged 15–49 years who were successfully tested for HIV infection as part of the 2010 RDHS. These women constituted a nationally representative sample of women who were 15 to 49 years old and residing in Rwanda during the 2010 RDHS interviews.

### Ethics statement

This study does not involve any experiment or interaction with human or animal subjects. It uses secondary data from the 2010 RDHS. The 2010 RDHS protocol for the blood specimen collection and testing for HIV was reviewed and approved by the Rwanda National Ethics Committee, the Institutional Review Board of ICF International, and the Centres for Disease Control and Prevention (CDC) in Atlanta.

### Analytic methods

#### Dependent variable

The outcome variable is the HIV-1 test result which was either positive (coded with 1) or negative (coded with 0). Other possible results such as indeterminate were not observed, leading to a binary response variable.

#### Covariates

Unlike other infectious diseases the HIV/AIDS incubation period is longer and varies across infected persons. The most successful models of the spread of HIV have been mostly based on underlying and proximate risk factors in describing the risk of infection from infected to susceptible individuals [16,19,36,37].

The proximate-determinants conceptual framework for integrating demographic and epidemiological approaches to research on HIV [28] played a reference role in the selection of potential demographic, socio-economic, cultural and proximate factors that were used as explanatory variables. The existing literature and factors available from the 2010 RDHS also guided the selection of covariates.

The candidate predictor variables identified from the 2010 RDHS include underlying factors namely the *woman’s age*, *sex of household head*, *age of household head*, *woman’s mobility (away for one month or more in last 12 months)*, *marital status*, *religion*, *household’s economic status*, *type of place of residence*, *woman’s education*, *woman’s occupation*, *woman’s exposure to mass media*, *HIV/AIDS stigma*, *and knowledge-perception of HIV/AIDS*. Selected proximate determinants include the *age at first sex*, *number of sexual partners in addition to husband/regular partner within last 12 months (recent concurrent sexual partner)*, *recent sexually transmitted infections (within preceding 12 months)*. *The District of residence* is included to assess probable clustering in HIV test result data. This is modelled as district-level spatial effects. Most of the covariates were recoded to be comparable with the existing studies [19,20,31]. In the context of Rwanda, the household headship becomes an important factor that can explain the spread of HIV following the four-year war that culminated in the 1994 Genocide perpetrated against Tutsi where many adult persons, particularly men, were killed.

Economic status represents the level of the household’s wealth index. The second and the third quintiles were grouped with the middle into a “medium” household’s economic status. The wealth index provides an aggregate measure of the distribution of wealth across respondents’ households as it combines information about the household assets, source of drinking water, sanitation, and house characteristics. Only education and employment are not included [38]. Exposure to mass media is measured in the 2010 RDHS using 3 indicators: (i) “*Frequency of reading newspapers or magazines*”, (ii) “*Frequency of listening to radio*”, and (iii) “*Frequency of watching television*” [8]. The woman’s exposure status index was constructed following the classification proposed by Fazle Rabbi [39] as the highest value of the four indicator values. Therefore, the exposure index was categorised as “not exposed” (0 exposed to none), “moderate exposure” (maximum = 1), and “high exposure” (2 and above).

Principal component analysis was used to construct the HIV stigma and knowledge-perception indices [40,41]. The HIV/AIDS *stigma index* was created using four indicators for the respondents’ accepting attitudes. These were framed as their willingness to (i) take care of a family member with HIV at home, (ii) buy fresh vegetables from a vendor who has HIV, (iii) allow a HIV-positive female teacher to continue teaching, and (iv) let others know the HIV status of family members who have HIV [8]. The stigma index was then classified as ‘low’, ‘medium’, or ‘high’ [41].

The HIV *knowledge-perception* i*ndex* was constructed based on the woman’s answers to five indicator questions which asked the respondent whether: (i) a healthy-looking person can have and transmit HIV; (ii) the AIDS virus can be transmitted by mosquito bites; (iii) a person can become infected with HIV by sharing food with someone who is infected; (iv) people can reduce their chances of getting the AIDS virus by using a condom every time they have sex, and (v) people can get the AIDS virus because of witchcraft or other supernatural means [8]. The index was also categorised into ‘low’, ‘moderate’, or ‘high’ levels [41]. An event is referred to as ‘recent’ if it occurred 12 months preceding the 2010 RDHS.

#### Statistical analysis

A binary response variable is typically modelled using a binary logistic regression model [32,42]. The latter offers advantages over other models, such as the probit model, in terms of easier interpretation of analytical results [24,43]. A univariate standard logistic regression model was used to test for the association of each single covariate with the outcome variable. The association was considered statistically significant at 5% level of significance.

Covariates that have a statistically significant association with the HIV test result were then included in a multivariable analysis based on a geoadditive logit regression model. Data management and univariate analysis were carried out using IBM SPSS Statistics for Windows version 20.0 and multivariable analysis was conducted using BayesX version 2.1, the software for Bayesian inference in structured additive regression models [43].

Let *Y*_*is*_ denotes the binary outcome variable taking on the value “HIV positive”, coded with 1, with an unknown probability *p*. If the set of covariates contains *δ* categorical covariates *L*, and *v* continuous covariates *X*, then the geo-additive logit model for the woman *i*residing in district *s*; *s* = 1, …, 30 being more likely tested HIV positive is given by equation (1)[22,32].

$$\mathrm{logit}(p_{is})=\beta_{0}+\beta_{1}l_{i1}+\cdots +\beta_{\delta }l_{i\delta }+f_{1}(x_{i1})+\cdots +f_{\nu }(x_{i\nu })+f_{spat}(District_{s})$$ (1)

This class of models and its advantages over the classical models have been discussed in details elsewhere [20,31,32,43,44]. In the left side of equation (1), *p*_*is*_ = *E*[*Y*_*is*_] represents the probability of woman *i* from district *s* testing HIV positive, *logit*(.) is a logit link function with ${\mathrm{logit(p}}_{\text{is}}\text{)}=\text{ln}\left(\frac{p_{\mathrm{is}}}{1-p_{is}}\right)$. The right side of equation (1), *η*_*is*_ = β_0_ + β_*1*_ *l*_*i1*_ + … +β_*δ*_l_*iδ*_ + f_1_(x_*i1*_) + … + *f*_*v*_(*x*_*iv*_) + *f*_*spat*_(*District*_*s*_), is the geo- additive predictor where the parameter vector β = (β_0_, β_1_, … β_δ_)’ is the vector of linear fixed-effects of categorical covariates that are modelled parametrically. The functions *f*_*j*_(.), *j* = *1*, …, *v* represent nonlinear effects of metrical covariates on the outcome status. Thus continuous covariates are modelled non-parametrically making (1) a semi-parametric model. The district effect *f*_*spat*_(*District*_*s*_) represents the spatial effect of district-specific and random effects. It can therefore be subdivided into structured and unstructured (random) effect as shown in equation (2) [45].

$$f_{spat}(District_{s})=f_{struc}(District_{s})+f_{unstruc}(District_{s})$$ (2)

The inference is fully Bayesian and is based on MCMC simulations, thus all model parameters and variances are random variables. It follows that assumptions and prior distributions of the model parameters have to be specified.

For fixed-effect parameters *β* independent diffuse priors *π(β) ∝* constant are assumed. Typically inverse-Gamma *IG*(*a*_*j*_, *b*_*j*_) priors (3) are assigned to all unknown variances $\tau_{j}^{2}$ [46] where constant parameters *a_j_* > 0 and *b_j_* > 0, and *a_j_ = b_j_* = 0.001 is a standard choice for a weakly informative prior.

$$\pi \left(\tau_{j}^{2}\right)\propto \frac{1}{{\left(\tau_{j}^{2}\right)}^{a_{j}+1}}\exp\left(-\frac{b_{j}}{\tau_{j}^{2}}\right)$$ (3)

Nonparametric effects *f*_*j*_(.) of metrical covariates are modelled as Bayesian P-splines as proposed by Lang and Brezger [47]. They are specified through Bayesian penalized splines (P-splines) with partially improper random walk priors for the B-spline coefficients. In effect the classical approximation (4) of the function *f*_*j*_(*x*_*j*_) as a linear combination of B-splines basis function *B*_*m*_ of degree *q* defined over a grid of *k* + 1 equally spaced knots [47]:

$$\hat{f}j\left(x_{j}\right)=\sum_{m=1}^{d_{j}}\gamma_{jm}B_{m}\left(x_{j}\right)\;\;,d_{j}=q+k$$ (4)

has its Bayesian analog first- or second-order random walk smoothness priors

$$\gamma_{jm}=\;\gamma_{j,m-1}+u_{jm}\;or\;\gamma_{jm}=2\gamma_{j,m-1}+u_{jm}$$ (5)

with independent and identically distributed Gaussian errors $u_{jm}\sim N\left(0,\tau_{j}^{2}\right)$ and diffuse, locally constant priors *π(γ*_*j1*_ *)∝* constant or *π(γ*_*j1*_) and *π(γ*_*j2*_ *)∝* constant for initial values [46,48]. Second-order random walk smoothness priors and cubic splines were considered in this study data analysis.

The structured spatial components of *f*_*spat*_ *(District*_s_), were modelled through Markov random field (MRF) prior as a spatial index of neighbouring districts was defined instead of exact spatial coordinates of the woman’s residence [43,46]. We assumed that districts are neighbours if they share boundaries and the random effects of a district *s* to be conditionally Gaussian, with the mean of the effects of neighbouring areas as expectation and the variance that is inversely proportional to the number of neighbours *n*_*s*_ of district *s*. That is,

$$f_{spat}\left(s|s^{\prime }\right)=\beta_{s}^{spat}|\beta_{s^{\prime }}^{spat},s\neq s^{\prime }\sim N\left(\frac{1}{n_{s}}\sum_{s^{\prime }\in \delta_{s}}\beta_{s^{\prime }}^{spat},\frac{\tau_{spat}^{2}}{n_{s}}\right)$$ (6)

Where *s*’ ∈ *δ*_*s*_ denotes that district *s*’ is a neighbour of district *s*. The amount of spatial smoothness was controlled by the variance $\tau_{spat}^{2}$.

It was assumed that all priors for parameters were conditionally independent and all priors were mutually independent. Sensitivity analysis to the choice of prior parameters were carried out with different pairs of parameters *a* and *b*. Common values of the hyper parameters are *a* = *b* = 0.00001, *a* = 1, *b* = 0.005, and *a* = *b* = 0.00001 [43,46,49]. The latter is the default in BayesX 2.1 and it was used in the analysis of this study data. In general, with well-identified parameters and large sample sizes, reasonable choices of prior distributions have minor effects on posterior inferences [46].

#### Model selection

According to proximate-determinants conceptual framework [28], underlying factors operate through proximate determinants in order to affect the status of health outcomes. These factors are, given the selected factors, clustered into (i) context-related underlying factors: sex of household’s head; woman’s age; marital status; religion; place of residence; household’s economic status; woman’s occupation, (ii) intervention program-related underlying factors: HIV-related stigma; and HIV-related knowledge-perception indices), and proximate determinants which include recent STIs, presence of recent sexual partners excluding husband or regular partner, and the age at first sexual intercourse.

Univariate standard logistic regression models were used to identify factors that were individually associated with the HIV test result. After the univariate analysis, this study conducted a series of geoadditive multiple logistic regression analyses. The spatial effects were investigated first. Thereafter, the estimation of more complex models was performed according to the clusters of the selected risk factors. All categorical covariates were dummy coded and the first factor levels, as presented in Table 2, were considered as reference categories. The following six models were fitted. The short forms used to represent long-name variables that might not be self-explanatory include *EcoStatus* for level of household economic status, *MStatus* for woman’s marital status, *Age* for woman’s current age, *Place* for type of place of residence, *Knowledge* for HIV knowledge-perception, *Ageatsex* for woman’s age at first sex, and *Concurrent* for recent concurrent sexual partners.

**Table 2:** Distribution of HIV infection and test of its association with selected factors.

Determinants (missing data)	Levels	Frequency (percent)*		P-value
		**HIV negative**	**HIV positive**	
**Underlying determinants**				
*Woman’s age*	Continuous	27.95 (9.57)	33.56 (8.51)	<0.001
*Age of household head*	Continuous	42.72 (13.63)	42.92 (12.52)	0.808
*Sex of household head*	Male	4457 (64.11)	124 (1.78)	<0.001
	Female	2229 (32.06)	142 (2.04)	
*Woman’s marital status*	Lives in union	3308 (47.58)	127 (1.83)	<0.001
	Never in union	2739 (39.40)	51 (0.73)	
	No longer in union	639 (9.19)	88 (1.26)	
*Mobility (Number of trips last 12 months)*	Continuous	1.19 (5.42)	1.18 (3.12)	0.970
*Woman’s religion* (0.17%)	Christian	6490 (93.52)	244 (3.52)	<0.001
	Muslim	78 (1.12)	12 (0.17)	
	Other	107 (1.54)	9 (0.30)	
*Type of place of residence*	Urban	1114 (16.02)	102 (1.47)	<0.001
	Rural	5572 (80.15)	164 (2.36)	
*Economic status*	Low	1195 (17.19)	40 (0.58)	<0.001
	Medium	3960 (56.96)	112 (1.61)	
	High	1531 (22.02)	114 (1.64)	
*Woman’s educational level* (0.03%)	No education	982 (14.13)	45 (0.65)	0.080
	At most primary	4578 (65.85)	165 (2.37)	
	Beyond primary	1126 (16.20)	56 (0.81)	
*Woman’s occupation* (0.17%)	Agriculture	4231 (60.97)	142 (2.05)	0.001
	Other	2444 (35.22)	123 (1.77)	
*Woman currently employed* (0.09%)	No	1908 (27.47)	78 (1.12)	0.788
	Yes	4772 (68.70)	188 (2.71)	
*Exposure to mass media* (0.12%)	Not exposed	421 (6.07)	13 (0.19)	0.646
	Low	1492 (21.51)	61 (0.88)	
	High	4758 (12.02)	63 (2.75)	
*Stigma index* (0.09%)	Low	3510 (50.53)	246 (2.02)	<0.001
	Moderate	1636 (23.55)	106 (1.53)	
	High	1534 (22.08)	20 (0.29)	
*HIV knowledge-perception index* (0.36%)	Low	1745 (25.19)	50 (0.72)	<0.001
	Medium	4063 (58.65)	195 (2.82)	
	High	855 (12.34)	19 (0.27)	
**Proximate determinants**				
*Age at first sex (0*.*04%)*	< 19 years	1696 (24.41)	110 (1.58)	<0.001
	Other	4987 (71.77)	156 (2.24)	
*Recent STIs (0*.*16%)*	No	6307 (90.87)	203 (2.92)	<0.001
	Yes	368 (5.30)	63 (0.91)	
*Recent concurrent sexual partners*	None	6336 (91.14)	225 (3.24)	<0.001
	One or more	350 (5.03)	41 (0.59)	

* For continuous covariates the mean and standard deviation are presented

*Model1*:*η*_***1***_ ***=*** *fstr(District)* **,** fitted unadjusted structured district-level spatial effect.

*Model2*:*η*_***2***_ ***=*** *f*_*struc*_ *(District)* ***+*** *f*_*unstruc*_ *(District*,), fitted unadjusted total district-level spatial effect.

$$\begin{array}{l} Model\;3:\begin{array}{l} {\text{η}}_{\text{3}}={\text{β}}_{\text{0}}+f\left(\mathrm{Age}\right)+{\text{β}}_{\text{1}}\mathrm{Sex}+{\vec{\beta }}_{\text{2}}\mathrm{MStatus}+{\vec{\beta }}_{\text{3}}\mathrm{Religion}+{\text{β}}_{\text{4}}\mathrm{Place}+{\vec{\beta }}_{\text{5}}\mathrm{EcoStatus}\\ \;\;\;\;\;\;\;+{\text{β}}_{\text{6}}\mathrm{Occupation}+f_{\mathrm{struc}}\mathrm{(District)}+f_{\mathrm{unstruc}}\mathrm{(District)} \end{array} \end{array}$$

Model 3 adjusted spatial effects for all underlying determinants. The latter include context- and intervention program- related risk factors. The corresponding geoadditive predictor is given as: ($\vec{\beta }$ indicates that more than one parameter were estimated for the concerned factor according to the number of corresponding dummy variables).

The form of the woman’s age effect was found to be nonlinear at exploratory analysis. Hence, the woman’s age was modelled as a continuous covariate.

*Model4*: *n4 = β1Concurrent + βAgeatsex + β3STIs + f*_*struc*_ *(District) + f*_*unstruc*_ *(District)*

Explored the extent to which proximate determinants explain the risk of HIV infection.

*Model5*: Spatial effect was adjusted for both underlying and proximate determinants. The geoadditive predictor is written as

$$\begin{array}{l} \eta_{\text{5}}={\text{β}}_{\text{0}}+\text{f}\left(\text{Age}\right)+{\text{β}}_{\text{1}}\text{Sex}+{\vec{\beta }}_{\text{2}}\text{MStatus}+{\vec{\beta }}_{\text{3}}\text{Religion}+{\text{β}}_{\text{4}}\text{Place}+{\vec{\beta }}_{\text{5}}\text{EcoStatus}\\ \;\;\;\;\;\;\;\;\;+{\text{β}}_{\text{6}}\text{Occupation}+{\vec{\beta }}_{\text{7}}\text{Stigma}+{\vec{\beta }}_{\text{8}}\text{Knowledge}+{\text{β}}_{\text{9}}\text{Concurrent}+{\text{β}}_{\text{10}}\text{Ageatsex}+{\text{β}}_{\text{11}}\text{STIs}\\ \;\;\;\;\;\;\;\;\;+{\text{f}}_{\text{struc}}\text{(District)}+{\text{f}}_{\text{unstruc}}\text{(District)} \end{array}$$

*Model6*: This model fitted nonlinear effects and fixed-effects present in Model 5 only, leaving out spatial effects. This model assessed the extent to which the presence of spatial components improved other parameter estimates. The corresponding additive predictor is written as

$$\begin{array}{l} {\text{η}}_{\text{6}}={\text{β}}_{\text{0}}+\text{f}\left(\text{Age}\right)+{\text{β}}_{\text{1}}\text{Sex}+{\vec{\beta }}_{\text{2}}\text{MStatus}+{\vec{\beta }}_{\text{3}}\text{Religion}+{\text{β}}_{\text{4}}\text{Place}+{\vec{\beta }}_{\text{5}}\text{EcoStatus}\\ \;\;\;\;\;\;\;\;\;+{\text{β}}_{\text{6}}\text{Occupation}+{\vec{\beta }}_{\text{7}}\text{Stigma}+{\vec{\beta }}_{\text{8}}\text{Knowledge}+{\text{β}}_{\text{9}}\text{Concurrent}+{\text{β}}_{\text{10}}\text{Ageatsex}+{\text{β}}_{\text{11}}\text{STIs}. \end{array}$$

In particular, Model 1 was fitted to examine geographical variation in HIV infection among women due to district-specific characteristics without taking into account any other risk factor. Model 2 investigates potential differences in HIV infection due to both district-level random and structured spatial effects.

For each fitted model, inference was based on Markov Chain Monte Carlo simulation techniques. The results are based on 1000 samples generated with 12000 iterations, 2000 burn-ins by taking every 10^th^ sample. The estimated geoadditive models were compared based on the Deviance Information Criterion (DIC) values which provide a measure of goodness-of-fit for comparing nested Bayesian models especially when the latter contain nonparametric and random effects. The smaller the DIC, the better the fit [50].

## Results

### Women profile and univariate analysis

In total, of 6952 women who provided their blood samples for HIV testing, 266 (3.8%) were HIV positive. The average age of sampled women was 33.56 years and 27.95 years for HIV-positive and HIV-negative women respectively. Table 2 presents the frequency distribution of both infected (prevalence) and uninfected women according to the selected HIV infection risk factors. For continuous covariates the mean and standard deviation are presented. The p-values for the tests for associations of each selected risk factor and the HIV test result are presented in the last column of Table 2.

The univariate variable selection procedure suggested that the *sex of the household head*, *woman’s age*, *marital status*, *religion*, *household’s economic status*, *place of residence*, *woman’s occupation*, *HIV knowledge-perception*, *HIV/AIDS-related stigma*, *recent occurrence of sexually transmitted infections (STIs)*, *age at first sexual intercourse*, *recent concurrent sexual partners* had a statistically significant association with the woman’s HIV test result.

According to the results in Table 2, the highest missing data is on the HIV-knowledge-perception index where it is as low as 0.36%. In total 79 cases representing 1.13% of the sample had missing data on at least one of the factors and they were not considered in the subsequent multivariate data analysis. This rate of missing data is low enough (less than 5%) to be inconsequential [51,52]. We decided to conduct a complete-case data analysis. The cases with missing data on one of the significant factors were dropped from multivariate analysis. The studied sample then reduced to 6873 women, of whom 261 (3.8%) were HIV positive. The following results are based on the reduced sample for which all cases have complete data on each of the risk factors considered for multivariate regression analysis.

### Results of geoadditive modelling of HIV infection

The assessment and selection of the model which provides a better data fit was based on the DIC values presented in Table 3. The measure of model complexity (pD) and the posterior mean deviance $\bar{D}$statistics are also reported. Model 5 with the smallest DIC was preferred, although the improvement over Model 6 is quite small (difference in DIC = 1.24). Model 5 is also a very significant improvement of all other Models, with the differences in DIC statistics that are significantly higher than 7 [50]. In addition Model 5 enables an assessment of spatial clustering in HIV test result data that would not be possible with Model 6. It follows that the data interpretation and discussion are based on the results obtained from Model 5.

**Table 3:** Models comparison based on the DIC statistic.

Statistic	Model 1	Model 2	Model 3	Model 4	Model 5	Model 6
DIC	2163.98	2163.22	1929.66	2039.68	1860.59	1861.83
pD	18.37	20.45	25.87	23.10	29.38	20.66
$\bar{D}$	2127.24	2122.33	1877.92	1993.47	1801.83	1820.52

#### Unadjusted spatial effects

**Fig 2:**
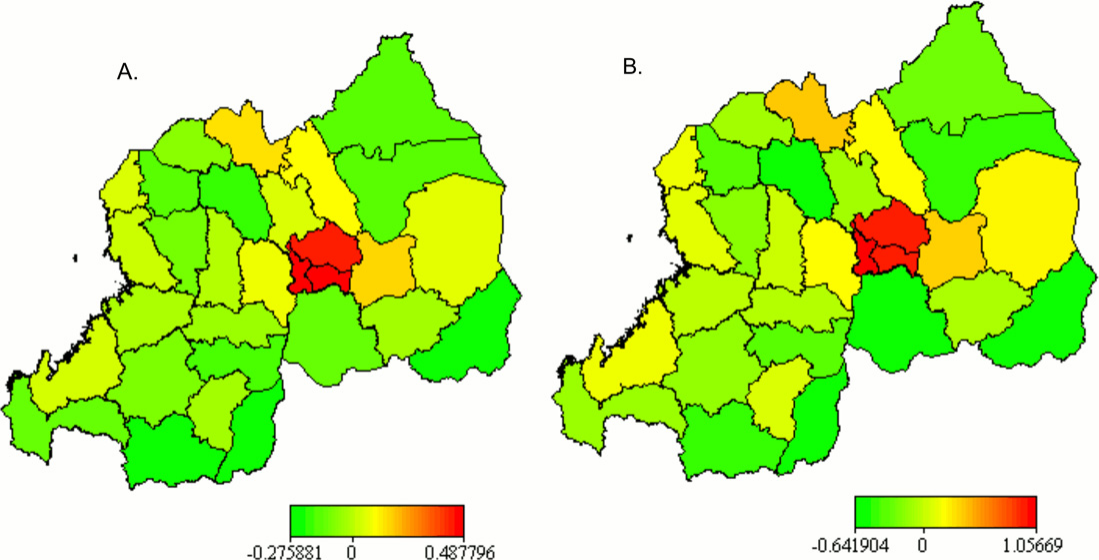
Posterior mean of the unadjusted structured (A) and total (B) spatial effects.

The maps of the unadjusted posterior mean of district-level structured (A) and total (B) spatial effects (based on Model 2) in Fig. 2 show that, without taking into account the underlying and proximate determinants of HIV infection, the risk of infection was highest in the three districts of the City of Kigali. Unstructured spatial effect map (not shown here) also shows similar pattern. There appeared particular geographic patterns of HIV infection. For example, the districts lying along Lake Kivu (Rubavu, Rutsiro, Karongi and Nyamasheke) presented a moderate risk of HIV infection. A moderate risk is also visualised for all districts neighbours of the City of Kigali (with the exception of Bugesera), for Burera district connecting to Uganda, and for Kayonza which shares border with Tanzania. The three districts of the City of Kigali were associated with higher risk of HIV infection. District-level unadjusted structured (Fig. 2A) and total (Fig. 2B) spatial effect maps are practically similar to the map of the HIV prevalence rate among adult women in Fig. 1.

**Fig 3:**
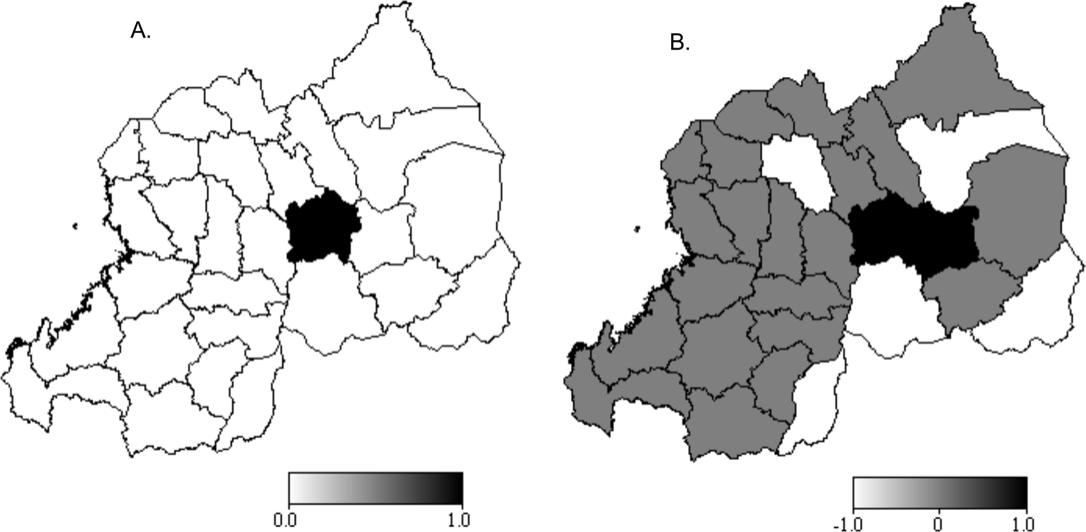
Maps of 95% (A) and 80% (B) posterior probabilities for the unadjusted total spatial effects.

The 95% (A) and 80% (B) posterior probability maps for the unadjusted total spatial effect, in Fig. 3, show a significant difference in district level spatial effects on HIV test result. The 95% probability map on Fig. 3A shows that all districts of the City of Kigali are significantly associated with a higher (black colour) risk of HIV infection (credible intervals strictly positive), and that all other districts are not statistically associated (white colour) with the risk of HIV infection. The 80% posterior probability map on Fig. 3B shows however that Rwamagana, Nyarugenge, Kicukiro and Gasabo districts are associated with a significantly higher (black colour) risk of infection, while Gatsibo, Bugesera, Gakenke, Kirehe and Gisagara districts are associated with a significantly lower (white colour) risk of HIV infection. There is no statistically significant (grey colour) spatial effect on the risk of HIV infection associated with the other 21 districts.

#### Adjusted effects

Model 5 adjusts spatial effects with the selected underlying and proximate determinants of HIV infection. With the same model the proximate risk factors’ effects are also adjusted with the selected underlying factors. Thus, Model 5 allows the assessment of adjusted spatial effects and the identification of the key determinants of HIV infection simultaneously. In the same modelling process the effect of each underlying determinant that is not mediated through proximate and spatial effects is estimated.

#### Fixed and nonlinear effects

Table 4 presents the results for fixed-effects parameter estimates based on Model 5. The posterior odds ratios (POR), and corresponding 95% credible interval (CI) limits (derived from the 2.5% and 97.5% quantiles of the posterior mean estimates of linear effects) are indicated. Categorical covariates with statistically significant association with the risk of HIV infection include all selected proximate determinants (age at first sex, recent STIs, and recent concurrent sexual partners), HIV/AIDS-related stigma, household economic status, type of place of residence, marital status, and sex of household head.

**Table 4:** Posterior odds ratio (POR) estimates for fixed effects with 95% credible intervals based on Model 5.

Variable (reference category)	POR	95% CI for POR
Constant	0.022	(0.012,0.040)
*Sex of household head* (Male)	1.000	
Female	1.793	(1.267,2.537)*
*Marital status* (Lives in union)	1.000	
Never in union	0.851	(0.524,1.370)
No longer in union	2.067	(1.392,3.043)*
*Religion* (Christian)	1.000	
Muslim	1.670	(0.815,3.445)
Other	1.354	(0.583,2.918)
*Place of residence* (Urban)	1.000	
Rural	0.755	(0.621,0.927)*
*Economic status* (Low)	1.000	
Medium	1.135	(0.766,1.704)
High	1.737	(1.084,2.835)*
*Woman’s occupation* (Agriculture)	1.000	
Other	1.255	(0.885,1.790)
*Stigma index* (Low)	1.000	
Medium	1.742	(1.330,2.323)*
High	0.434	(0.262,0.703)*
*Knowledge-perception index* (Low)	1.000	
Moderate	1.362	(0.988,1.950)
High	0.913	(0.502,1.600)
*Recent concurrent sexual partners* (None)	1.000	
One or more	1.669	(1.093,2.573)*
*Age at first sex* (< 19 years)	1.000	
Other	0.666	(0.506,0.884)*
*Recent STIs* (No)	1.000	
Yes	3.881	(2.737,5.366)*

*Statistically significant different PORs

The results suggest that women living in a female-headed household or in a household with a high economic status are associated with a higher risk of being tested HIV-positive compared with those living in male-headed or low economic status households. This is based on the corresponding PORs of 1.793 with 95% CI: (1.084, 2.835); and 1.737 with 95% CI: (1.084, 2.835) respectively. The 95% CI does not contains 1 indicating that the corresponding fixed-effects are statistically significant. After controlling for the effect of other socioeconomic, demographic factors and spatial effects, the difference in the risk of HIV infection among women residing in rural [POR = 0.755 with 95% CI: (0.621, 0.927)] and urban areas remains statistically significant. The odds of a woman being HIV-infected are more than 24% lower in rural than in urban areas. Similarly, women who lived in a sexual relationship (with a regular partner or husband) but were no longer in such a union (widow, divorced or separated) [POR = 2.067; 95% CI: (1.392, 3.043)] were more than two times more likely to be HIV infected compared with those who were living with their husbands or regular partners. As expected those women who had never lived in a sexual union appeared to be at a lower, but statistically insignificant, risk of HIV infection [POR = 0.851; 95% CI: (0.524, 1.370)] than those who were living in a regular union.

Similarly, it appeared that women whose occupation is other than agriculture, run more than 25% higher odds of being HIV infected (although statistically insignificant) than any other woman. Compared to Christians, the risk of HIV infection is higher among Muslim women [POR = 1.670; 95% CI: (0.815, 3.445)].

Regarding intervention program-related factors, evidences showed that women with a high HIV knowledge-perception index are associated with a statistically insignificant reduced risk of HIV infection [POR = 0.913; 95% CI: (0.502, 1.600)] than women with a low index. However, those women with a low knowledge-perception index are better off than those with a moderate index. Unexpectedly, women with high HIV/AIDS stigma showed a statistically significant low risk of testing HIV-positive [POR = 0.434; 95% CI:(0.262,0.703)] than their counterparts who reported a low HIV/AIDS stigma. However, this should be interpreted with caution as the situation is opposite for women with a medium HIV stigma index [POR = 1.362, 95% CI: (0.988, 1.950)]. Notwithstanding these numerical values, the data show that HIV/AIDS-related stigma is a significant and complex factor of HIV infection among women in Rwanda.

The results also show that all selected proximate determinants have a statistically significant effect on the HIV test result. There is a highly significant association of being HIV positive and the recent occurrence of a sexually transmitted infection (STI) 12 months before the survey [POR = 3.881; 95% CI: (2.737,5.366)]. Women who had a recent STI were about four times more likely to be HIV positive than those who had not any. A statistically significant high risk of infection is associated with having one or more sexual partner in addition to a husband or regular partner [POR = 1.669; 95% CI: (1.093, 2.573)]. The age the woman initiated sexual intercourse remained a statistically significant risk factor for HIV infection after adjusting for all risk factors. Women whose age at first sex was 19 years or above experienced more than 30% lower odds of contracting HIV [POR = 0.666; 95% CI: (0.506,0.884)] than their counterparts whose age at first sex was less than 19 years.

**Fig 4:**
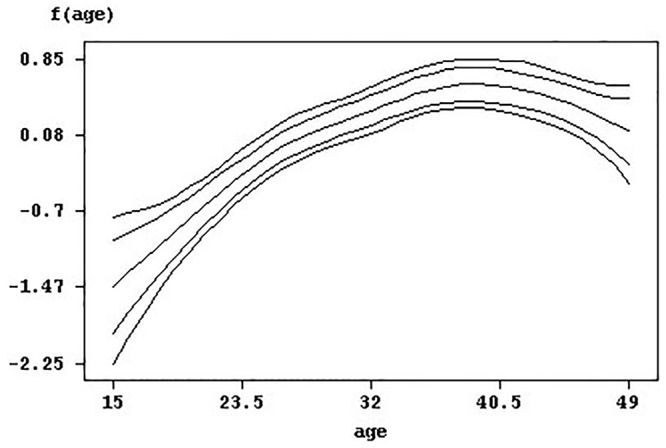
Effect of woman's age with pointwise 95% and 80% credible intervals.

Younger women aged 15–25 years were at a significantly lower but sharply increasing risk of infection. Fig. 4. depicts the nonlinear effects of the woman’s age on the risk of infection along with 95% and 80% credible intervals. The significantly positive and moderately increasing effect attenuates until around the age of 46 years when it levels off and declines slowly, becoming statistically insignificant until the age of 49 years where the 95% credible interval widens and limits have opposite signs. In summary, the woman age’s effect on the risk of HIV infection has approximately an oblique inverse U-shape.

#### Spatial effects

**Fig 5:**
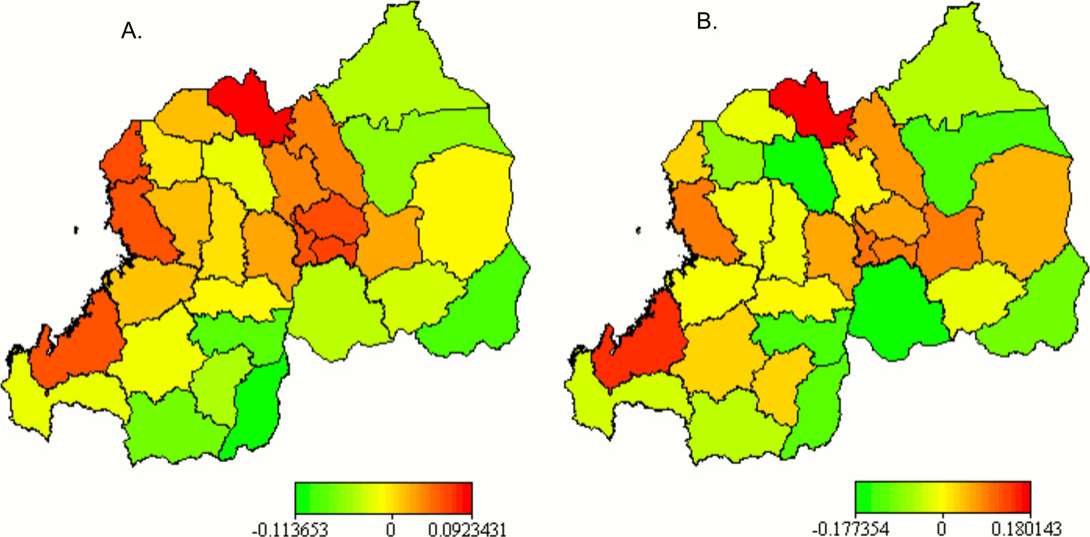
Posterior mean of the adjusted structured (A) and unstructured (B) spatial effects.

The posterior mean spatial effects estimated with Model 5 are presented in Fig. 5 for the structured (A) and unstructured (B) effects. The map of the total spatial effect pattern is very similar to the unstructured effect map and is not shown here. The 95% and 80% posterior probability maps (not displayed) indicated that there was no statistically significant association of district-level spatial characteristics and the woman’s HIV test result. This suggests that there might be a strong relationship between modelled covariates and localised spatial processes that would differently expose resident women to HIV infection. The small difference in DIC between Model 6 and Model 5 (presented in Table 5) also evidences a strong association of the covariates and the district-specific spatial factors.

**Table 5:** Sensitivity analysis: DIC, posterior mean variance, its standard error (within brackets), and 95% CI based on Model 5.

Effects	a = 0.00001, b = 0.00001	a = 0.001, b = 0.001	a = 0.01, b = 0.01
$f(age):\tau_{Age}^{2}$	0.011 (0.019)	0.013(0.020)	0.025(0.038)
**2.5%–97.5%**	0.0007–0.0527	0.0012–0.0609	0.0035–0.1210
$\tau_{struc}^{2}$	0.041 (0.097)	0.075 (0.128)	0.1101(0.141)
**2.5%–97.5%**	0.00001–0.3333	0.0005–0.4590	0.0042–0.5254
$\tau_{unstruc}^{2}$	0.034 (0.056)	0.051 (0.061)	0.0704(0.0629)
**2.5%–97.5%**	0.00001–0.1873	0.0007–0.2200	0.0052–0.2382
DIC	1861.72	1860.59	1860.19

Nonetheless, there are distinctive geographic patterns in both structured and unstructured district-level spatial effects visualised in Fig. 5. Districts located in the western and central to northern regions of Rwanda are highly affected by structured spatial effect, while the eastern and southern regions show lower structured spatial effects. The western region consisting of districts located along Lake Kivu (with exception of Rusizi) namely Rubavu, Rutsiro, Karongi and Nyamasheke districts show a higher association with the risk of HIV infection. Similarly, are associated with a higher risk of HIV infection the districts of Nyarugenge, Kicukiro and Gasabo districts, which constitute the City of Kigali (largely urban and semi-urban), and its 4 neighbouring districts, namely Kamonyi to its west, Rwamagana to its east, and Rulindo and Gicumbi to its north. Burera district which is the neighbour of both Rulindo and Gicumbi to their north and sharing a border with Uganda is also associated with a higher risk of infection. This pattern has almost an inverse T-shape with the centre of the arm situated in the City of Kigali and the top of the stem being Burera district. The data show that the districts that share borders with Burundi and Tanzania are associated with a lower risk of HIV infection. The majority of them are in the Southern Province.

### Sensitivity analysis

The results of the sensitivity analysis of model estimates to the choice of hyper-parameter values for Model 5 are presented in Table 5. The estimated model parameters do not largely depend on the choice of hyper-parameter values. Relatively large modifications in the values of hyper-parameters, a and b, are associated with small variations in the posterior mean variances of the nonlinear and spatial effects. The standard choice of *a* = *b* = 0.001 which is the default for BayesX 2.1 was used to analyse this study data with geoadditive Model 5.

## Discussion

This study is the first statistical mapping of HIV infection among women in Rwanda that is based on nationally representative and population-based data. In addition, this study uses a data driven unified and advanced modelling framework to simultaneously assess the effects of both key risk factors and spatial processes susceptible of explaining HIV infection differentials [32,43,49]. While there has been a strong desire for such studies in various countries [20,31], the strength of this study lies in its capability to match individual women’s HIV test results with the corresponding women’s data from the 2010 RDHS. The matched data provided rich information about potential HIV risk factors.

This study shows that women members of women-headed households, and those who were no longer in their former sexual union are associated with a higher risk of HIV infection. The increased risk of HIV infection among divorced, separated and widowed, and the protective effect of living with a regular sexual partner have been reported elsewhere [53]. However, it is difficult to establish whether this is due to their sexual behaviour or HIV infection among their former partners.

There was a significant difference in the risk of infection between rural and urban women that was not mediated through spatial effects. The risk is higher among women residing in urban areas than those residing in rural areas. This has been often reported for other communities [19]. It is argued that in urban areas there is an increased freedom for sexual networking, relaxed attachment to cultural values compounded with a highly diversified and dynamic migration that fuels the spread of HIV [15,17,54]. Similarly the economic status improvement parallels increasing risk of infection. Unlike the reported narrowing gap in the risk of HIV infection in South Africa between wealthier and poorest economic classes [19], there is a significant difference between high and low economic status women in Rwanda. However, the difference between the medium and low economic status women is quite small.

A number of studies reported that educational level and age are associated with the risk of HIV infection [13,19,29]. This study found that HIV infection among women increases with age and declines beyond 45 years. On the contrary, the data used in this study could not establish any statistical association between woman’s educational attainment and the HIV test result.

The results indicate that the risk of HIV infection among women with a high index of HIV stigma is significantly lower compared with those with a low index. There was however inconsistency in the direction of such protective effect. Women with a low stigma index were better off than those with a medium stigma. This indicates that this factor is more complex and that the result should be carefully interpreted. An explanation is that the influence of the HIV stigma level (as well as HIV-knowledge-perception) continuously competes with and can be cancelled out by other factors such as gender inequality, cultural norms and traditions about sex, and poverty. In such situations, the choice to have sex, to have safer sex, or delay first sex is not a decision over which most women have control [29,55,56]. It has been found that HIV stigma is influential for both HIV testing and HIV test result disclosure [26,30,41,57].

Particular attention should be paid to women in occupations other than agriculture as they are more likely to be HIV-infected, and residents of urban areas [17]. In fact, these women constitute an important part of the skilled labour force at higher risk of HIV infection.

Sexually transmitted infections constitute a very strong risk factor of HIV infection among women of reproductive age in Rwanda. Women who reported recent STIs were highly associated with the positive HIV test result. It is very important to note that an occurrence of STIs carries double the risk of HIV acquisition. It not only exposes the woman to an increased risk of infection, but she may also infect her sexual partners with STIs, rendering them more susceptible to HIV infection. The role of STIs in increasing HIV risk has been documented by other studies [27,58,59].

According to the patterns of structured spatial effect, the risk of HIV infection is associated with living in the 3 Districts of the City of Kigali, namely Nyarugenge, Kicukiro and Gasabo, and in four neighbouring Districts including Rwamagana to the east, Kamonyi to the west, and Rulindo and Gicumbi to the north. Burera District, which is the northern neighbour of the latter two districts, is also associated with a higher risk of infection. The reason this rural district is associated with higher spatial effects can be linked with its position in relation to the Volcanoes National Park. The HIV might be largely transported through movements of national and international tourists who are mostly attracted by mountain gorillas. In addition it might also be brought across the border from neighbouring Uganda, where HIV prevalence rates are higher [2,4]. Socio-cultural practices such as polygamy in this region might be another local risk factor to explain this result.

The second high risk region consists of districts lying along Lake Kivu. It includes Rubavu, Rutsiro, Karongi and Nyamasheke districts. The intense movement between the towns of Goma in the DRC and Gisenyi in Rubavu District, many tourism sites and hotels on the Lake Kivu shore, and many fishery zones where mostly fishermen spend weeks and even months away from their families, may explain higher HIV infections in this region. It was also reported that fishermen’s wives in Kenya were more mobile than their husbands and were associated with a higher HIV prevalence [60]. The association of mobility within and across country borders and increased HIV transmission have also been reported elsewhere [15,61].

Mobility can also partially explain the statistically insignificant differences in district-level spatial effects in Rwanda whereby well-maintained road networks throughout the country potentially expose low and neighbouring high prevalence areas to an equal risk of HIV infection. In fact, in high prevalence areas HIV awareness, and knowledge about HIV prevention and transmission becomes relatively higher than in low prevalence areas.

In summary, the mapping of HIV infection and the identification of key background and proximate risk factors is crucial for the design of prioritised HIV intervention programs that are responsive to district-specific characteristics including geographic differences. The evidence suggests that HIV interventions would achieve a significant outcome among women in Rwanda if they focus on changing their sexual behaviours. In addition, family-centred interventions would yield positive outcomes, especially if women-headed households are targeted. Sexuality education programs, including late sex debut, and safe sex with a single partner should also be targeted by HIV intervention programs in Rwanda. In addition to prevention, timely diagnosis and treatment of sexually transmitted infections can constitute a very important component of the action against HIV infection. Furthermore, district-specific characteristics should be taken into account while designing HIV interventions. Most importantly, the usual concentration of HIV intervention programs within high prevalence areas would not lead to significant reduction in HIV infection if neighbouring low prevalence areas, where HIV awareness is generally lower, are not equally targeted.

### Study limitations

Some covariates in the same cluster have a certain level of correlation which can reduce the precision in the estimated effects. In addition, the construction of a factor index might affect the estimated association of concerned covariates with the dependent variable. This study is also based on a cross-sectional survey whereby some questions are answered retrospectively. Not only causality cannot be established, but also such questions require the respondent to recall how an event happened in the past, such as concurrent sexual partnerships and occurrence of sexually transmitted infections. In addition, due to disclosure problems associated with moral and ethical values, the corresponding cases are more likely to be underreported. This limitation was, to a certain extent, attenuated by only considering events which occurred in the 12 months preceding the survey. However, this may also lead to an underestimation of these factors’ effect on the risk of HIV infection which might have occurred more than one year before the survey.

Notwithstanding these limitations, the results of this study remain valid and have significant policy implications for the prevention of HIV spread among women of child bearing age in Rwanda.

## Conclusion

The aim of this study was to investigate district-level geographic variation of HIV infection and to identify key risk factors among women of the child bearing age in Rwanda. The study successfully applied a unified Bayesian structured geoadditive modelling methodology on matched HIV test results and other women’s data from the 2010 RDHS. The district-level structured, random and total spatial effects, nonlinear effects of continuous covariates as well as the fixed effects of categorical covariates were estimated.

All selected proximate determinants remained statistically significant risk factors after adjusting for underlying factors. Residing in women-headed and high-economic status households, in urban areas, or being separated, divorced or widowed were significantly associated with an increased risk of HIV infection. Geographic patterns of district-level spatial effects were also highlighted on visual maps. Thus, this study provides pragmatic information required for improved policy making that pertains to controlling HIV infection in Rwanda.

The district-level HIV prevalence rate is a contextual proximate determinant which is an average of the dependent variable. It can reflect district-specific characteristics which are partly modelled as structured spatial effects. Including HIV prevalence in the geoadditive predictor would provide insight into the risk of HIV infection when every woman is equally exposed to infected persons [28]. Further studies could explore this possibility. Furthermore, the HIV/AIDS stigma’s effect suggests further research into the interaction effect, mediated through HIV/AIDS stigma, of socio-cultural and behavioural factors on the risk of HIV infection among women in Rwanda.

## References

[1] UN. The Millennium Development Goals Report 2011. New York: United Nations Department of Economic and Social Affairs (DESA), 2011.

[2] UNAIDS. Global report: UNAIDS Report on the Global AIDS Epidemic 2010: WHO Library Cataloguing-in-Publication Data; 2010.

[3] UNAIDS. Global report: UNAIDS Report on the Global AIDS Epidemic 2012: WHO Library Cataloguing-in-Publication Data; 2012.

[4] UNAIDS. Global report: UNAIDS Report on the Global AIDS Epidemic 2013: WHO Library Cataloguing-in-Publication Data; 2013.

[5] NISR, MOH, Macro International Inc. Rwanda Service Provision Assessment Survey 2007. Calverton, Maryland, U.S.A.: NISR, MOH, and Macro International Inc., 2008.

[6] UNAIDS/WHO. Epidemiologic fact sheet on HIV/AIDS and sexually transmitted infections: 2000 update, Rwanda. Geneva: 2000.

[7] INSR, ORC Macro. Rwanda Demographic and Health Survey 2005. Calverton, Maryland, U.S.A: INSR and ORC Macro, 2006.

[8] NISR, MOH, ICF International. Rwanda Demographic and Health Survey 2010. Final Report. Calverton, Maryland, USA: NISR, MOH, and ICF International 2011.

[9] WHO. PMTCT strategic vision 2010–2015: preventing mother-to-child transmission of HIV to reach the UNGASS and Millennium Development Goals. WHO Library Cataloguing-in-Publication Data 2010 10.1016/S0140-6736(14)60844-8

[10] Villamor E, Misegades L, Fataki M, Mbise R, Fawzi W. Child mortality in relation to HIV infection, nutritional status, and socio-economic background. International Journal of Epidemiology. 2005;34:61–8. 15649965 10.1093/ije/dyh378

[11] Nakiyingi JS, Bracher M, Whitworth JAG, Ruberantwari A, Busingye J, Mbulaiteye SM, et al Child survival in relation to mother's HIV infection and survival: evidence from a Ugandan cohort study. AIDS. 2003;17:1827–34. 12891069 10.1097/00002030-200308150-00012

[12] Mmbaga EJ, Hussain A, Leyna GH, Mnyika KS, Sam NE, Klepp K-I. Prevalence and risk factors for HIV-1 infection in rural Kilimanjaro region of Tanzania: Implications for prevention and treatment. BMC Public Health. 2007;7(58):1–9. 17445264 10.1186/1471-2458-7-58 PMC1866238

[13] Cogneau D, Grimm M. Socioeconomic status, sexual behaviour, and differential AIDS mortality: evidence from Cote d’Ivoire. Popul Res Policy Rev. 2006;25:393–407.

[14] Jesmin S, Chaudhuri S, Abdullah S. Educating women for HIV prevention: does exposure to mass media make them more knowledgeable? Health Care Women Int 2013;34(3–4):303–31. 10.1080/07399332.2012.695829 23394327

[15] Kishamawe C, Vissers DCJ, Urassaa M, Isingo R, Mwaluko G, Borsboom GJJM, et al Mobility and HIV in Tanzanian couples: both mobile persons and their partners show increased risk. AIDS. 2006;20(4):601–8. 16470125 10.1097/01.aids.0000210615.83330.b2

[16] Johnson K, Way A. Risk factors for HIV infection in a national adult population: Evidence from the 2003 Kenya Demographic and Health Survey. Journal of Aquired Immune Deficiency Syndromes. 2006;42(5):627–36. 10.1097/01.qai.0000225870.87456.ae 16868500

[17] Orubuloye IO, Caldwell P, Caldwell JC. The role of high-risk occupations in the spread of AIDS: Truck drivers and itinerant market women in Nigeria. International Family Planning Perspectives. 1993;19(2):43–8+71.

[18] Hargreaves JR, Bonell CP, Boler T, Delia Boccia, Birdthistle I, Fletcher A, et al Systematic review exploring time trends in the association between educational attainment and risk of HIV infection in sub-Saharan Africa. AIDS. 2008; 22:403–14. 10.1097/QAD.0b013e3282f2aac3 18195567

[19] Bärnighausen T, Hosegood V, Timaeus IM, Newell M-L. The socioeconomic determinants of HIV incidence: evidence from a longitudinal, population-based study in rural South Africa. AIDS. 2007;21(Suppl 7):S29–S38. 10.1097/01.aids.0000300533.59483.95 18040162 PMC2847257

[20] Wand H, Whitaker C, Ramjee G. Geoadditive models to assess spatial variation of HIV infections among women in Local communities of Durban, South Africa. International Journal of Health Geographics. 2011;10(28):1–9. 10.1186/1476-072X-10-28 21496324 PMC3098769

[21] Glynn JR, Caraël M, Auvert B, Kahindo M, Chege J, Musonda R, et al Why do young women have a much higher prevalence of HIV than young men? A study in Kisumu, Kenya and Ndola, Zambia. AIDS. 2001;15(Suppl 4):S51–S60. 11686466 10.1097/00002030-200108004-00006

[22] Ngesa O, Mwambi H, Achia T. Bayesian spatial semi-parametric modeling of HIV variation in Kenya. PLoS ONE. 2014;9(7):e103299 10.1371/journal.pone.0103299 25061669 PMC4111556

[23] Chimoyi LA, Musenge E. Spatial analysis of factors associated with HIV infection among young people in Uganda, 2011. BMC Public Health. 2014;14(1):555. 24898872 10.1186/1471-2458-14-555 PMC4061924

[24] Adebayo SB, Fakolade R, Anyanti J, Ekweremadu B, Ladipo O, Ankomah A. Modelling level, trend and geographical variations in stigma and discrimination against people living with HIV/AIDS in Nigeria. SAHARA J: journal of Social Aspects of HIV/AIDS Research Alliance / SAHARA, Human Sciences Research Council. 2011;8(3):115–27. 10.1080/17290376.2011.9724994 23237726

[25] Betsi NA, Koudou BG, Cissé G, Tschannen AB, Pignol AM, Ouattara Y, et al Effect of an armed conflict on human resources and health systems in Cote d’Ivoire: Prevention of and care for people with HIV/AIDS. AIDS Care. 2006;18(4):356–65. 16809113 10.1080/09540120500200856

[26] Goudge J, Ngoma B, Manderson L, Schneider H. Stigma, identity and resistance among people living with HIV in South Africa. Journal of Social Aspects of HIV/AIDS. 2009;6(3):94–104. 20485849 10.1080/17290376.2009.9724937

[27] Cohen MS. Sexually transmitted disease enhance HIV transmission: no longer a hypothesis. Lancet 1998;351(Suppl III):5–7. 10.1016/s0140-6736(98)90002-2 9652712

[28] Boerma JT, Weir SS. Integrating demographic and epidemiological approaches to research on HIV/AIDS: The Proximate-determinants framework. The Journal of Infectious Diseases. 2005;191(Suppl 1):S61–7. 15627232 10.1086/425282

[29] Burgoyne AD, Drummond PD. Knowledge of HIV and AIDS in women in sub-Saharan Africa. African Journal of Reproductive Health. 2008;12(2):14–31. 20695040

[30] Yang H, Li X, Stanton B, Fang X, Lin D, Naar-King S. HIV-related knowledge, stigma, and willingness to disclose: A mediation analysis. AIDS Care. 2006;17(7):717–24. 10.1080/09540120500303403 PMC1933389 16971280

[31] Kandala N, Campbell E, Rakgoasi D, Madi B. The geography of HIV/AIDS prevalence rates in Botswana. HIV/AIDS—Research and Palliative Care. 2012;4:95–102. 10.2147/HIV.S30537 PMC3411371 22870041

[32] Kammann EE, Wand MP. Geoadditive models. Applied Statistics. 2003;52(Part 1):1–18.

[33] GoR. Organic Law N° 29/2005 of 31/12/2005 determining the administrative entities of the Republic of Rwanda. Official Gazette of the Republic of Rwanda. 2005.

[34] NISR, MINECOFIN. Rwanda Fourth Population and Housing Census 2012. Thematic Report on Population size, structure and distribution. National Institute of Statistics of Rwanda, 2014.

[35] NISR. Rwanda Statistical Yearbook 2012. National Institute of Statistics of Rwanda, 2012.

[36] Lemme F, Doyle AM, Changalucha J, Andreasen A, Baisley K, Maganja K, et al HIV infection among young people in northwest Tanzania: The Role of biological, behavioural and socio-demographic risk factors. PLoS ONE. 2013;8(6):e66287 23805209 10.1371/journal.pone.0066287 PMC3689734

[37] McIntosh WA, Thomas JK. Economic and other societal determinants of the prevalence of HIV: A test of competing hypotheses. The Sociological Quarterly. 2004;45(2):303–24.

[38] Rutstein SO, Johnson K. The DHS Wealth Index DHS Comparative Reports No. 6. Calverton, Maryland: ORC Macro, 2004.

[39] Rabbi AMF. Mass Media Exposure and its Impact on Fertility: Current Scenario of Bangladesh. Journal of Scientific Research. 2012; 4(2):383–95.

[40] Vyas S, Kumaranayake L. Constructing socio-economic status indices: how to use principal components analysis. Oxford University Press 2006:459–8. 10.1093/heapol/czl029 17030551

[41] Achia TNO, Obayo E. Trends and correlates of HIV testing amongst women: lessons learnt from Kenya. African Journal of Primary Health Care & Family Medicine. 2013;5(1):Art. #547, 10pages.

[42] Hastie T, Tibshirani R. Generalized additive models for medical research. Statistical Methods in Medical Research. 1995;4:187 8548102 10.1177/096228029500400302

[43] Belitz C, Brezger A, Kneib T, Lang S. BayesX: Software for Bayesian Inference in Structured Additive Regression Models Version 2.0.1 Methodology manual 2012 Available from: http://www.stat.uni-muenchen.de/~bayesx/manual/methodology_manual.pdf.

[44] Kandala N-B, Fahrmeir L, Klasen S, Priebe J. Geo-additive models of childhood undernutrition in three Sub-Saharan African countries. Popul Space Place. 2009;15: 461–73.

[45] Lawson AB. Bayesian disease mapping Hierachical modeling in spatial epidemiology. New York: CRC Press, Chapman & Hall Taylor & Francis Group; 2009.

[46] Fahrmeir L, Kneib T. Propriety of posteriors in structured additive regression models: Theory and Empirical Evidence. Journal of Statistical Planning and Inference. 2008; 139:843–59.

[47] Lang S, Brezger A. Bayesian P-Splines. Journal of Computational and Graphical Statistics. 2004;13 (1):183–212.

[48] Hennerfeind A, Held L, Sauleau EA. A Bayesian analysis of relative cancer survival with geoadditive models. Statistical Modelling. 2008;8(2):117–39.

[49] Brezger A, Kneib T, Lang S. BayesX: Analyzing Bayesian Structured Additive Regression Models. Journal of Statistical Software. 2005; 14(11):1–22.

[50] Spiegelhalter DJ, Best NG, Carlin BP, Linde AVD. Bayesian measures of model complexity and fit. Journal of the Royal Statistical Society: Series B (Statistical Methodology). 2002; 64 (4):583–639.

[51] Schafer JL. Multiple imputation: a primer. Statistical Methods in Medical Research. 1999;8:3–15. 10347857 10.1177/096228029900800102

[52] Dong Y, Peng C-YJ. Principled missing data methods for researchers. SpringerPlus. 2013;2(222):1–17. 10.1186/2193-1801-2-222 23853744 PMC3701793

[53] Kalichman S, Ntseane D, Nthomang K, Segwabe M, Phorano O, Simbayi L. Recent multiple sexual partners and HIV transmission risks among people living with HIV/AIDS in Botswana. Sex Transm Infect 2007;83(5):371–775. 17475684 10.1136/sti.2006.023630 PMC2659030

[54] Voeten HACM, Vissers DCJ, Gregson S, Zaba B, White RG, Vlas SJd, et al Strong Association Between In-Migration and HIV Prevalence in Urban Sub-Saharan Africa. Sex Transm Dis. 2010;37(4):240–3. 10.1097/OLQ.0b013e3181c3f2d0 19959971 PMC3514976

[55] Maharaj P, Cleland J. Condom use within marital and cohabiting partnerships in KwaZulu-Natal, South Africa. Studies in Family Planning. 2004;35(2):116–24. 15260213 10.1111/j.1728-4465.2004.00013.x

[56] Pettifor AE, Rees HV, Kleinschmidt I, Steffenson AE, MacPhail C, Hlongwa-Madikizela L, et al Young people's sexual health in South Africa: HIV prevalence and sexual behaviors from a nationally representative household survey. AIDS. 2005;19:1525–34. 16135907 10.1097/01.aids.0000183129.16830.06

[57] Reniersa G, Eatond J. Refusal bias in HIV prevalence estimates from nationally representative seroprevalence surveys. AIDS. 2009;23(5):621–9. 10.1097/QAD.0b013e3283269e13 19182677 PMC2695508

[58] Catherine A. Hankinsa, Friedman SR, and TaZ, Strathdeee SA. Transmission and prevention of HIV and sexually transmitted infections in war settings: implications for current and future armed conflicts. AIDS. 2002;16(17):2245–52. 12441795 10.1097/00002030-200211220-00003

[59] Røttingen J, Cameron D, Garnett G. A systematic review of the epidemiological interactions between classic sexually transmitted diseases and HIV: how much is really known? Sexually Transmitted Diseases. 2001;28(10):579–97. 11689757 10.1097/00007435-200110000-00005

[60] Kwena ZA, Camlin CS, Shisanya CA, Mwanzo I, Bukusi EA. Short-term mobility and the risk of HIV infection among married couples in the fishing communities along Lake Victoria, Kenya. PLoS ONE. 2013;8(1):e54523 10.1371/journal.pone.0054523 23336005 PMC3545885

[61] Cassels S, Manhart L, Jenness SM, Morris M. Short-term mobility and increased partnership concurrency among men in Zimbabwe. PLoS ONE. 2013;8(6):e66342 23824635 10.1371/journal.pone.0066342 PMC3688871

